# Evaluation of a Transition to University Programme for Students with Autism Spectrum Disorder

**DOI:** 10.1007/s10803-018-3776-6

**Published:** 2018-10-12

**Authors:** Jiedi Lei, Steph Calley, Mark Brosnan, Chris Ashwin, Ailsa Russell

**Affiliations:** Centre for Applied Autism Research, Department of Psychology, University of Bath, Bath, BA2 7AY UK

**Keywords:** Autism Spectrum Disorder, University, College, Students, Transition, Intervention

## Abstract

Applying to university can be an anxiety-provoking time for many autistic students, though enrolment can be increased by actively involving them in transition planning. We provide an evaluation of a transition to university pilot programme (Autism Summer School) for autistic students (16–19 years) who are seeking to apply/attend university. The content focused on introducing students to various aspects of university life including academic (sample lectures), social (e.g., clubs and societies), and daily living (eating in university canteen and staying in student accommodation). Students’ quantitative and qualitative feedback are positive and promising, showing significant reduction across a range of concerns related to transition to university after the programme, as well as general optimism related to starting university.

The transition from secondary to tertiary or higher education (such as university) is not always easy for many students who aspire to attend post-secondary education. Beyond the educational challenge of entry requirements lies a need for students to develop levels of independence in managing accommodation, finances, catering, and meeting increased academic demands as compared to secondary school (high school). It has long been argued that building social networks represents one of the major challenges as the transition from school to university can involve leaving home, family, and friends, and represent a large shift socially for many young people, especially in their first year of study (Fisher and Hood 1987). This first year transition typically requires students to become socially and academically integrated and has been operationalised by the following definition of psychosocial wellness in students as:

Equipped to handle the demands for independent functioning that accompany the college transition, including developing an academic schedule, negotiating a new and often complex social world, and developing the internal motivation to wake up at a reasonable time, attend classes and keep up with assignments (Mattanah et al. 2004, p. 213).

Many of the academic, social, and daily living challenges can seem magnified for student with Autism Spectrum Disorder (ASD) making this transition. ASD is a neurodevelopment condition characterised by difficulties in social communication and interaction, and a restricted, repetitive, or stereotyped pattern of behaviours, interests, and activities (American Psychiatric Association 2013). In addition to the core characteristics of Autism, up to 70% of children and young people also experience co-occurring mental health conditions such as anxiety (Simonoff et al. 2008; Steensel et al. 2011; White et al. 2009) and depression (Wigham et al. 2017). In addition, other common occurring difficulties include hypersensitivity to sound and light (Longtin 2014; Sarrett 2018), and poor executive functioning (Demetriou et al. 2018; Ozonoff et al. 1991). It may be that this myriad of challenges faced by autistic students^1^ have a significant impact on students’ transition and successful adjustment to university life (Gelbar et al. 2014). For example, social communication deficits may mean the more complex social environment at university is difficult to navigate (Gobbo and Shmulsky 2014; Longtin 2014). A preference for routine, certainty, and other facets of the repetitive behaviours domain in autism may also mean that students are ill-equipped to adjust to the changes in everyday routines and living required (Barnhill 2016; Geller and Greenberg 2009). Furthermore, executive function impairments may also suggest that future oriented flexible planning, time management, and organisational skills may be particularly problematic for a large proportion of autistic young people (Barnhill 2016; Dipeolu et al. 2014). Consequently, it is important for autistic students seeking to apply to university to consider the issue of whether or not to disclose their diagnosis, as well as when and to whom they should disclose, which can influence access to various types of support available at university which may ameliorate some of these issues (Adreon and Durocher 2007; Dipeolu et al. 2014; Fleury et al. 2014).

Recent reports suggest that although around half (46%) of autistic students have the intellectual potential (Centers for Disease Control and Prevention 2012) to attend higher education (Sanford et al. 2011), the number of enrolment, retention, academic achievements, and employment rate following graduation is still relatively lower compared to students with other disabilities, as well as their typically developing peers (AGCAS Disability Task Group 2014; Gobbo and Shmulsky 2014; Lucas and James 2018). A recent report (Jackson et al. 2018) also found in a sample of 56 autistic adults currently enrolled in post-secondary institutions in the US, UK, and Canada, around three quarters reported feelings of isolation, high levels of stress, anxiety, depression, and suicidal ideation, further highlighting the poor state of mental health for many autistic students when transitioning to university. Furthermore, completing post-secondary education often did not serve as a direct stepping stone for many autistic students, as participation in competitive part/full-time employment is relatively poor. In the UK, autistic students are twice as likely to be unemployed compared to students with other disabilities within 6 months of graduating from university, and four times more than typically developing peers (AGCAS Disability Task Group 2014). It is therefore important for autistic students, family members and professionals to understand that while accessing higher education may confer many life benefits beyond attaining a degree level qualification, a university degree alone is not a guarantee of post university employment or occupational success. Given the potential additional complexity of challenges faced by autistic students, it is essential for university stakeholders and transition services to be aware of the potential needs of autistic students, to facilitate preparation for university life, and to provide informational, practical, and emotional support during the transitional process. In a longitudinal study of 830 autistic students (Chiang et al. 2012), successful transition planning was found to be one of the strongest predicting factors of attending university.

To date, reports of interventions to support autistic students in making a successful transition to first year of university have included contacts (e.g., Shmulsky et al. 2015) and campus-based events for students considering and seeking to apply to university (i.e., pre-transitional) (e.g., Retherford and Schreiber 2015), as well as provision of peer support during the early stages of the actual transition (e.g., Littlefield 2010). Although research has been relatively scarce in this area, there is some evidence to indicate that pre-transitional interventions predict the decision to attend university (Chiang et al. 2012; Wei et al. 2016). Chiang et al. (2012) found that 61% students who participated in transition planning and 74% of students who received instruction specifically focused on transition planning participated in postsecondary education. This participation in postsecondary education rate is almost two to three times greater compared to students who did not participate in transition planning (36%) or receive transition-planning instructions (24%). The content of pre-transitional interventions reflects the challenges associated with autism described above, such as social skills and social relationships, practical and emotional support, organizational difficulties such as time-management and problem-solving skills, sensory challenges and mental health concerns (Cai and Richdale 2016; Camarena and Sarigiani 2009; Cox et al. 2017; Mitchell and Beresford 2014; Van Hees et al. 2015; White et al. 2016).

The content of pre-transitional interventions can be informed by a number of qualitative studies, which have explored the areas of concern for autistic students prior to college and university attendance. Studies have included participants from secondary education settings, current university students, students attending transition programmes, and support groups. Common themes emerging from qualitative studies have included coursework requirements, disability awareness on campus, social skills and social relationships, availability of practical and emotional support, organisational difficulties such as time-management and problem-solving skills, sensory challenges and mental health concerns (Cai and Richdale 2016; Camarena and Sarigiani 2009; Mitchell and Beresford 2014; Van Hees et al. 2015; White et al. 2016). Wider concerns have also been raised such as disability awareness on campus and issues around disclosing diagnosis (Camarena and Sarigiani 2009; Cox et al. 2017).

Thus, developing well-informed and useful pre-transition packages and transition packages may improve outcomes amongst young autistic adults attending university. Relevant outcomes can be conceptualised as short-term outcomes, e.g., reduced worries about university and increased confidence about transitioning; medium term outcomes (e.g., improved rates of university enrolment, student retention and completion); and long-term outcomes (e.g., improved earnings across the lifespan and greater life satisfaction). The present study describes the short-term outcomes of a pre-transitional programme in the form of a summer school for autistic students wishing to attend University. The programme aimed to provide direct experiences of university life and thus supported exposure to the focus of commonly reported worries and concerns about university transition. Consecutive delivery of the programme over the course of 5 years allowed student feedback and evaluation to shape each subsequent iteration of the programme, and will discuss some of the findings from the pilot transitional programme for autistic students wishing to attend University. We hypothesized that students may experience fewer concerns about university transition following participating in the autism summer school, as the programme hopes to reduce uncertainty related to various aspects of daily, academic, and social life at university.

## Methods

### Participants

A total of 125 students participated in the Autism Summer School programme from 2013 to 2017, of whom 122 students gave consent to take part in research evaluation. All participants applied for the [Bath] Autism Summer School through the online application form hosted on the university’s website. Eligibility was assessed on the following criteria: (1) having a prior diagnosis of Autism, Asperger’s, Pervasive Developmental Disorder—Not Otherwise Specified (PDD-NOS), or Autism Spectrum Disorder from a clinical professional; (2) 16 years or older; (3) with a planned or actual application to go to university for a 3–4 year undergraduate degree programme. A total of up to 30 places were offered on a first come first serve basis to participants each year. Prior to taking part in the autism summer school, all parents and students were given an information sheet to read about the autism summer school, and asked to consent for whether they would like to take part in the research evaluation of the autism summer school. Participants who did not give consent for research but were offered a place on the programme were still able to attend the autism summer school, though we were unable to use the information they provided as part of the research evaluation. The current study is in line with the Declaration of Helsinki (revised in 2000) and was approved by the university’s departmental ethics committee.

### Pre-transitional Programme: Autism Summer School

The autism summer school comprised two overnight stays in student accommodation at a campus university, and a curriculum delivered across 3 days. The curriculum was designed to prepare autistic students for typical university life and promote self-care and wellbeing when at university. Upon arrival, each student received a package containing a detailed timetable and brief outline of session contents, as well as printed session hand-outs. Sessions were structured across three strands ‘work’, ‘rest’, and ‘play’. All sessions were delivered face-to-face in a group. The sessions taught included introducing students to the nature of academic life at university such as experiencing a typical lecture, socialising students to the role of staff and tutors, accessing student support and disclosing your diagnosis (‘work’ theme). There were also sessions about stress reduction, management of situational anxiety and the role of physical wellbeing with information about sporting facilities (‘rest’ theme). The strand of sessions about ‘play’ included information about clubs and societies at university, experiencing shared meals on campus, social outings and opportunities for informal socialising. The sessions were prepared and delivered by academic and clinical staff in the department of Psychology, or staff from the disability and careers service at the campus university, all of who have extensive experience working with autistic students at university. One session on students’ experience at university was delivered by autistic students either currently at or have recently graduated from either undergraduate or graduate studies. Clinical psychologists and clinical psychologists in training delivered the anxiety psycho-education session. Students were supported throughout the programme by ‘student ambassadors’, i.e., current university students trained to support widening participation and inclusion activities on campus. The student ambassadors received a specific training session in the needs of autistic students. None of the student ambassadors had a diagnosis of autism that we were aware about, but many had direct experience of working with autistic young people or direct personal experience of a family member on the spectrum.

The intervention was delivered on five separate occasions on consecutive years from 2013 to 2017. Evaluation was collected using mixed-methods, which focused on: (1) changes in short-term outcomes in respect of worries and concerns about attending university; (2) student satisfaction ratings with the overall programme; (3) qualitative written feedback about the summer school.

### Measures

#### Social Communication Questionnaire—Lifetime (Rutter et al. 2003)

The SCQ Lifetime is a 40-item parent-report measure that assesses the presentation of many autism symptoms, such as social communication difficulties, throughout the individual’s lifetime. Each item is scored using a dichotomous scale, where a score of “0” or “1” indicates the absence or presence of the symptom described, respectively. Further testing is recommended if the total score exceeds the cut-off of 15, which indicates the individual is likely to have a diagnosis of Autism Spectrum Disorder.

#### Transition to University Questionnaire (TUQ; Lambe et al. in press)

The TUQ is a 26-item self-report questionnaire developed during the first year of the Autism Summer School. Content was derived from the initial attendees (n = 25) at the Autism Summer School using a semi-structured questionnaire and focus groups, which lasted for 90 min (Lambe et al. in press). During the focus group, facilitators asked open questions such as: What worries you most about starting university? What part of university life are you looking forward to?. The themes that emerged from the thematic analysis were used to inform items on the TUQ. The items on the TUQ present a concern such as “when I think about going to university, I am concerned about the change in my routine”. Students are asked to rate each concern on a 5-point Likert scale from ‘strongly agree (5)’ to ‘strongly disagree (1)’. Individual item scores of > 3 (neither agree nor disagree) are taken to indicate endorsement of that concern.

### Student Satisfaction Feedback Questionnaire

Student satisfaction ratings were devised in collaboration with the Widening Participation team at the university. Widening Participation is a UK government initiative to help universities increase outreach and improve retention of students from disadvantaged backgrounds and students with disabilities. The feedback questionnaire asked students to rate on a 5-point Likert scale as to how enjoyable they found the autism summer school (1 = extremely unenjoyable); how helpful they found the autism summer school (1 = extremely unhelpful); and how positive or negative they felt about starting university (1 = extremely negative). Students were also asked to provide written feedback on: (1) what features of the autism summer school they found to be most helpful; (2) what they looked forward to about starting university.

### Study Design and Data Analysis

Every year, participants who consented were asked to fill out a series of demographics and the SCQ as part of participant characterisation to screen for autism symptom severity before arriving at the autism summer school. In years 2013–2017, participants were asked to complete the TUQ pre-arriving at the autism summer school. For years 2015–2017, participants were asked to re-rate at the end of the autism summer school only the concerns they had endorsed on the TUQ pre-arrival, and were also provided with space to identify any new concerns. Participants in 2013 did not complete the post-autism summer school TUQ as their data was used to pilot and tailor the questionnaire, rather than used for evaluation purposes. Student Satisfaction Feedback was collected for years 2013, and 2015–2017 on the last day of the autism summer school. Feedback for the second year (2014) was not collected during the autism summer school, as participants did not have time to complete feedback sheets due to shortage of time on the final day.

Data analyses are completed in five steps. In step one, using one-way ANOVAs and Bonferroni to correct for multiple comparisons, we characterised any differences across the five years in student demographics (age, gender, ethnicity, SCQ total score, and total TUQ score) to check whether any variables should be controlled for in subsequent analyses which pools data across the year groups, should significant differences arise between groups. We chose to conduct separate one-way ANOVAs analyses instead of a single one-way MANOVA as there were missing data for some of the participants across different dependent variables measured, and therefore constructing separate analyses allowed us to maximise sample size within each analyses. In step two, we assessed the different concerns and worries students reported using the TUQ pre-arriving at the autism summer school, and conducted an exploratory factor analysis to assess whether there are any domains of concerns that can be characterised at baseline. Identifying different domains of concerns enabled us to observe any changes in concerns post autism summer school at a more fine-grained level, rather than characterising changes in overall ratings of concerns.

Next, steps 3 and 4 were conducted using the TUQ pre- and post- autism summer school scores from years 2015–2017 as part of the evaluation of the effectiveness of the autism summer school in helping students reduce their concerns about going to university. In step three, we used one-way ANOVAs with Bonferroni to correct for multiple comparisons to assess whether there are any differences at baseline (pre-arriving at autism summer school) in the number of concerns endorsed, the total score of concerns, and the different subscale total scores based on the factors identified from step two between the year groups. This was to check if similar levels of concerns were endorsed across the different year groups. In step four, we assessed using paired sample T-tests whether any significant changes occurred in the scores of concerns endorsed reported at baseline, after the completion of the autism summer school, across all participants who completed the TUQ pre- and post-autism summer school.

In step five, we assessed the quantitative and qualitative feedback from the student satisfaction report from years 2013, and 2015–2017 to evaluate students’ perception of the summer school programme. For the quantitative feedback, we conducted a one-way MANOVA to assess whether students differed in their satisfaction of the programme across the years. For the qualitative feedback, we conducted a content analysis (Mayring 2015) where the first (JL) and last author (AR) independently formed coding templates which were used to code all the written feedback for each question. Any discrepancies that arose were resolved by discussing with the second author (MB) to reach agreement on developing a combined coding template. Using the final template, JL and AR each independently counted the frequency of quotes related to each category, and selected example illustrative quotes. Discrepancies were discussed with a final consensus reached. All statistical analyses were conducted using SPSS 22 (IBM), and Bonferroni was used to control for multiple comparisons where appropriate.

## Results

### Participant Demographics and Characterisation

Participant demographics and characterisation across each of the 5 years are shown in Table 1. A total of 122 participants took part across the years 2013–2017. Overall, the participants were aged between 16 and 30 years old (M = 17.76, SD = 1.56), 91 were male (74%), and largely Caucasian (90.1%). All the participants had received a formal diagnosis of ASD from a qualified clinician using DSM or ICD criteria. It was not possible to re-administer diagnostic assessments, however the parental reports on the SCQ were consistent with this group being characterised as autistic. Overall the SCQ scores were significantly above cut-off (≥ 15) [*t* (115) = 6.47, *p* = 0.001]. Across the years, participants did not show any significant differences in age [*F* (4,115) = 0.42, *p* = 0.79]; gender [*F* (4, 117) = 1.33, *p* = 0.26]; ethnicity [*F* (4, 116) = 0.93, *p* = 0.45]; or SCQ total [*F* (4, 111) = 2.25, *p* = 0.68). For years 2014–2017, participants did not show any differences in TUQ total concerns score pre-arriving at the autism summer school [*F* (3, 95) = 1.78, *p* = 0.16].

**Table 1:** Participant demographics and characterisation

	2013 (n = 22)	2014 (n = 22)	2015 (n = 26)	2016 (n = 31)	2017 (n = 21)
Age in years (M, SD)	18.05 (1.46)	17.45 (0.96)	17.84 (1.25)	17.77 (2.43)	17.67 (0.80)
Gender (n, %)					
Male	19 (86.4)	16 (72.7)	19 (73.1)	20 (64.5)	17 (81)
Female	3 (13.6)	6 (27.3)	7 (26.9)	9 (29)	4 (19)
Other	0	0	0	2 (6.5)	0
Ethnicity (n, %)					
Asian	1 (4.5)	0	1 (3.7)	1 (3.2)	0
Caucasian	21 (95.5)	21 (95.5)	22 (81.5)	26 (83.9)	19 (90.5)
Caribbean	0	0	0	1 (3.2)	0
Mixed	0	0	0	1 (3.2)	2 (9.5)
Other	0	1 (4.5)	3 (11.1)	1 (3.2)	0
Degree (n, %)					
Science/medicine	5 (22.7)	0	5 (18.5)	8 (25.8)	4 (19)
Tech/computing	2 (9.1)	0	4 (14.8)	3 (9.7)	2 (9.5)
Engineering	2 (9.1)	0	2 (7.4)	0	1 (4.8)
Maths	2 (9.1)	0	4 (14.8)	2 (6.5)	2 (9.5)
Languages	1 (4.5)	0	4 (14.8)	3 (9.7)	1 (4.8)
Social sciences	0	0	0	1 (3.2)	5 (23.8)
Arts/humanities	5 (22.7)	0	7 (25.9)	11 (35.5)	5 (23.8)
Unknown	5 (22.7)	22 (100)	0	2 (6.5)	1 (4.8)
SCQ	(n = 21)	(n = 19)		(n = 29)	
Total score (M, SD)	18.71 (5.78)	22.11 (6.93)	16.50 (8.10)	21.72 (8.59)	19.38 (7.53)
> Cut-off (15) (n, %)	17 (81)	17 (89.5)	15 (57.7)	22 (75.9)	15 (71.4)
TUQ baseline total					
M (SD)	NA	85.45 (17.59)	86.88 (15.26)	93.73 (16.79)	92.95 (11.98)
Range	NA	37–115	33–119	63–124	66–116

*SCQ* Social Communication Questionnaire Lifetime; *TUQ* Transition to University Questionnaire

### Transition to University Questionnaire—Exploratory Factor Analysis

We conducted an exploratory principal axis factor analysis using oblique rotation (direct oblimin) on the 26-items of the Transition to University Questionnaire, based on participants from 2014 to 2017’s responses before they arrived at the autism summer school (n = 98). The Kaiser–Meyer–Olkin measure verified the sampling adequacy for the analysis, KMO = 0.822 (Kaiser and Rice 1974), and all KMO values for individual items were > 0.64, which is above the acceptable limit of 0.5 (Kaiser and Rice 1974). An initial analysis was run to obtain eigenvalues for each factor in the data. Seven factors had eigenvalues over Kaiser’s criterion of 1 and in combination explained 56% of the variance. The scree plot also showed an inflexion after seven factors, justifying the retention of a seven-factor model. Table 2 shows the factor loadings after rotation. The items that cluster on the same factor suggest that Factor 1 represents a micro social world (developing friendships, fitting in, isolation, socialising); Factor 2 represents support (leaving friends, lack of support, course workload); Factor 3 represents macro social world (routine change, group work, accommodation, flatmates, bullied); Factor 4 represents leaving home (leaving home, leaving family, independence, coping alone, freshers’ week^2^); Factor 5 represents academic challenges (course difficulty, successful on course, enjoy course); Factor 6 represents daily living skills (cooking, getting lost, managing finances); and Factor 7 represents time management (managing time, meet course deadlines).

**Table 2:** Preliminary findings of factor loadings for Transition to University Questionnaire (n = 98)

	1	2	3	4	5	6	7
Develop friendships	**0.867**	− 0.038	0.124	0.042	− 0.07	− 0.086	0.077
Fitting in	**0.617**	0.078	− 0.032	− 0.091	0.016	0.134	− 0.053
Isolated	**0.564**	0.074	− 0.155	− 0.115	0.212	0.083	0.125
Socialising	**0.705**	− 0.047	0.136	− 0.234	− 0.155	0.046	− 0.071
Leaving friends	0.159	− **0.376**	0.004	− 0.042	0.308	0.13	0.17
Lack of support	0.215	**0.301**	0.003	− 0.034	0.107	0.149	0.107
Course workload	0.124	**0.585**	0.037	− 0.023	0.257	− 0.053	0.284
Changes in routine	− 0.215	0.008	**0.605**	− 0.222	0.014	0.227	0.092
Group work	0.127	− 0.09	**0.552**	− 0.097	0.001	0.091	− 0.169
Accommodation	− 0.03	0.052	**0.88**	− 0.171	− 0.085	− 0.219	0.12
Flatmates	0.363	− 0.07	**0.639**	− 0.025	0.046	− 0.131	0.201
Bullied	0.181	0.231	**0.477**	0.163	0.132	0.163	− 0.145
Leaving home	− 0.017	− 0.187	0.153	− **0.624**	0.263	− 0.027	0.037
Leaving family	0.086	− 0.068	0.127	− **0.668**	0.118	− 0.011	− 0.032
Independence	0.034	0.036	0.067	− **0.676**	− 0.113	0.052	0.114
Cope alone	0.181	0.187	− 0.035	− **0.613**	0.033	0.099	0.072
Freshers’ week	0.183	0.176	0.243	− **0.322**	0.268	0.065	− 0.06
Course difficulty	− 0.102	0.319	0.115	− 0.019	**0.567**	0.033	0.195
Successful on course	0.139	− 0.038	0.046	0.058	**0.667**	0.051	0.051
Enjoy course	− 0.098	0.037	− 0.064	− 0.159	**0.559**	− 0.035	− 0.044
Cooking	0.138	0.135	− 0.124	− 0.256	− 0.232	**0.421**	0.045
Getting lost	0.082	− 0.074	0.211	0.059	0.347	**0.497**	− 0.082
Finances	− 0.042	− 0.095	− 0.045	− 0.043	− 0.001	**0.724**	0.278
Manage time	− 0.003	− 0.067	0.023	− 0.049	− 0.053	0.09	**0.708**
Course deadlines	0.06	0.279	0.045	− 0.045	0.078	0.045	**0.635**
Food I like	0.24	0.202	0.137	− 0.117	− 0.034	0.292	− 0.13
Eigenvalues	7.98	2.25	2.02	1.67	1.38	1.15	1.11
% of variance	30.68	8.66	7.75	6.41	5.31	4.44	4.22
Cronbach’s alpha	0.825	0.426	0.822	0.836	0.66	0.608	0.706

Values highlighted in bold indicate questionnaire items that were selected for each factor

*M* mean; *SD* standard deviation; *Factor names 1* micro social world; *2* support; *3* macro social world; *4* leaving home; *5* academic issues; *6* daily living skills; *7* time management

### Characterising Participant Concerns About Going to University Before Summer School

Table 3 shows the characterisation of participants from 2015 to 2017’s concerns about going to university (measured by the TUQ). Overall, across all participants in 2015–2017 (n = 77), participants endorsed between 1 and 25 worries (M = 15.14, SD = 5.34), and the total concern score for the concerns endorsed ranged from 4 to 123 (M = 65.92, SD = 25.55). When all the items were included in the analysis and broken down into the seven factors identified from step two, the mean scores for each domain of concerns ranged from 3.24 to 3.90 (SD = 0.64 to 1.04). Participants did not show any differences in the number or total score of concerns they endorsed before coming to the autism summer school (*p* > 0.05). Using the preliminary factors identified from the TUQ in step 2, participants also did not show any differences in the average degree of concern across all the items within each subscale (rather than just concerns endorsed) (*p* > 0.05), with all average concern scores at baseline above 3 (neither agree nor disagree with the concern listed).

**Table 3:** Group differences in concerns associated with transitioning to university (Transitioning to University Questionnaire) prior to attending the Autism Summer School between 2015 and 2017

	M (SD)			F (2,74)	*p*
	2015 (n = 26)	2016 (n = 30)	2017 (n = 21)		
Endorsed concerns					
No. of items	13.88 (4.79)	15.63 (5.56)	16.00 (5.63)	1.12	.31
Total	59.35 (22.12)	69.33 (28.11)	69.19 (25.26)	1.31	.28
Factors (all items)					
1	3.35 (0.91)	3.89 (0.91)	3.80 (0.80)	2.91	.06
2	3.17 (0.73)	3.60 (0.78)	3.63 (0.64)	3.21	.05
3	3.62 (0.95)	3.74 (0.72)	3.64 (0.88)	.16	.86
4	3.30 (0.85)	3.51 (1.04)	3.54 (0.72)	.55	.58
5	3.26 (0.78)	3.67 (0.87)	3.54 (0.79)	1.78	.18
6	3.26 (0.94)	3.24 (0.79)	3.25 (0.93)	.00	.99
7	3.58 (0.95)	3.90 (0.84)	3.90 (0.66)	1.30	.28

*M* mean; *SD* standard deviation; *Factor names 1* micro social world; *2* support; *3* macro social world; *4* leaving home; *5* academic issues; *6* daily living skills; *7* time management

### Characterising Changes in Concerns About Going to University

Table 4 shows the mean scores for the concerns endorsed by all participants before arriving at the summer school across years 2015–2017, and the mean ratings of the same concerns after completing the autism summer school. Given that participants were only asked to re-rate the concerns they had endorsed pre-arriving at the autism summer school, not everyone completed all the items of the TUQ post-autism summer school, resulting in varying group sizes for each domain of concerns assessed. After using Bonferroni to correct for multiple comparisons, participants showed significant reductions in the total score for endorsed cocnerns, as well as reductions in each of the seven domains of concerns as identified by the TUQ factor analysis in step two following completion of the summer school (*p* < 0.001).

**Table 4:** Changes in endorsed concerns associated with transitioning to university pre and post Autism Summer School between 2015 and 2017

	Mean (SD)		Difference	95% CI	Cohen’s D	*df*	t	*p*
	Pre	Post						
Factors								
1 (n = 65)	4.33 (.37)	3.57 (.91)	.76	.54, .98	1.09	64	6.95	.00
2 (n = 64)	4.23 (.33)	3.46 (.74)	.76	.58, .94	1.34	63	8.40	.00
3 (n = 68)	4.38 (.38)	3.65 (.75)	.72	.56, .89	1.23	67	8.65	.00
4 (n = 64)	4.24 (.33)	3.51 (.90)	.72	.51, .93	1.08	63	6.92	.00
5 (n = 64)	4.24 (.37)	3.51 (.84)	.73	.52, .94	1.12	63	6.90	.00
6 (n = 59)	4.24 (.34)	3.58 (.80)	.66	.43, .88	1.07	58	5.84	.00
7 (n = 60)	4.36 (.42)	3.77 (.88)	.59	.34, .84	0.86	59	4.76	.00
Total (n = 77)	65.92 (25.55)	56.71 (26.60)	9.21	4.96, 13.46	0.35	76	4.32	.00

*M* mean; *SD* standard deviation; *Factor names 1* micro social world; *2* support; *3* macro social world; *4* leaving home; *5* academic issues; *6* daily living skills; *7* time management

### Student Satisfaction Feedback—Mixed Method Analyses

Table 5 shows the quantitative feedback provided by students from 2013 to 2015 to 2017 (n = 91) on the autism summer school programme, where no group differences were found across student ratings of enjoyableness and helpfulness of the autism summer school, as well as how positively they viewed going to university. Across all years, participants rated the autism summer school to be very enjoyable (M = 4.36, SD = 0.80), very helpful (M = 4.30, SD = 0.68), and viewed going to university somewhat positively (M = 3.93, SD = 0.96). These changes are significantly above the neutral value of 3 (*t*(91) = 9.20-18.37, *p* = 0.0001).

**Table 5:** Summary of quantitative student satisfaction feedback of Autism Summer School (n = 91)

	M (SD)				F (3, 87)	*p*
	2013 (n = 17)	2015 (n = 24)	2016 (n = 30)	2017 (n = 20)		
Enjoyable	4.35 (0.61)	4.33 (1.05)	4.43 (0.73)	4.30 (0.73)	0.14	0.94
Helpful	4.24 (0.56)	4.29 (0.75)	4.17 (0.53)	4.55 (0.83)	1.38	0.26
Starting University	4.18 (0.73)	4.08 (0.83)	3.80 (1.16)	3.79 (0.98)	0.90	0.47

*1* Least enjoyable/helpful/positive; *5* extremely enjoyable/helpful/positive

Of the 100 students who were asked to provide written qualitative evaluation, 96 students provided written feedback for the first question, and 70 students for the second question. Content analyses ascribed three codes for the question “What have you found to be most helpful at the Autism Summer School?” (see Table 6a). Although a small number (n = 3) of participants reported they did not find anything helpful about the autism summer school. However, the majority (n = 93) provided more detailed feedback, which was positive. First, many participants commented on both the content and delivery of the programme itself, especially about the various sample lectures the autism summer school had to offer, such as the psychoeducation about managing stress and anxiety, utilising their strengths at university, hearing experiences of past autistic students, diagnosis disclosure and accessing support at university, and learning about clubs and societies. Some of the participants commented:

**Table 6:** Summary of content analysis of qualitative student satisfaction feedback of Autism Summer School

Code	n (%)^a^
**(a) What have you found to be most helpful at the summer school? (n = 96)**	
*Programme*	*62 (64.6)*
Sample university lecture	5 (5.2)
Overall programme	5 (5.2)
Research	5 (5.2)
Psychoeducation	14 (14.6)
Academic strengths	7 (7.3)
Clubs and societies	11 (11.5)
Speakers with ASD	7 (7.3)
Disclosure/support	8 (8.3)
*Finding out about university life*	*33 (34.4)*
Accommodation	10 (10.4)
Meals	5 (5.2)
Campus tour	2 (2.1)
Subject specific activity	2 (2.1)
*Socialisation*	*22 (22.9)*
Typically developing student support	7 (7.3)
Meeting autistic students	6 (6.3)
Informal evening activities	4 (4.2)
*None of it/unsure*	*3 (3.1)*
**(b) What are you most looking forward about university? (n = 70)**	
*Education*	*19 (27.1)*
Course content	10 (14.3)
Studying	4 (5.7)
Academic achievement	4 (5.7)
*Independence/confidence*	*15 (21.4)*
*Practical daily living—accommodation*	*5 (7.1)*
*New beginnings*	*23 (32.9)*
New experiences	9 (12.9)
General optimism	14 (20)
*New social opportunities*	*39 (55.7)*
Meeting new people	20 (28.6)
Clubs and society	16 (22.9)
Autism identity	1 (1.4)
*Accessing support at university*	*2 (2.9)*
*None of it/unsure*	*4 (5.7)*

^a^Taken as % of students who provided written feedback for each question

The session on the systematic strengths of autism as this made me feel able to study at university and not just have social difficulties.

I enjoyed finding out about societies and clubs, and also how ASD traits are useful in life, both in study skills and in employment.

Second, some participants commented on the usefulness of finding out more about university life in general, such as experiencing staying in university accommodation, eating in canteens, as well as visiting the university campus and specific subjects’ department related to their interests. One participant commented:

The opportunity to stay in the halls as this helped me feel what university sleeping arrangements were like

Third, some participants commented on the social aspects of the summer school in general, and specifically about being able to meet both student ambassadors and other autistic students who are also transitioning to university, and having some informal evening social time. Some of the quotes on what participants found the be most helpful relating to social aspects included:

Meeting other prospective uni(versity) students who also had social difficulties and seeing that they could form friendships and care.

Being here among other autistic people with similar experiences and plans was helpful and also helped assuage my fears about being excluded and not fitting in as a mature student.

Content analysis ascribed six codes for the question “What are you most looking forward to about university?” (see Table 6b). Four participants did not comment on anything they were looking forward to at university, though the summer school experience has helped them to rethink about going to university:

After taking part in the summer school, I am going to think about my future to see if university is the right choice for me.

The majority of participants (n = 66) said they looked forward to going to university, the most popular theme was the new social opportunities that university had to offer (n = 39), and participants were generally looking forward to meeting new people through academic courses, accommodation, and joining new clubs and societies, as well as meeting other people with autism. Participants commented on looking forward to:

The opportunity to meet new people on my course or from clubs and societies.

I have a couple of new acquaintances that will be starting at the university with me, easing the potential social difficulties. I am more aware of what the uni(versity) has to offer socially.

Being part of a wider ASD community.

Second, many participants commented on their optimistic outlook on starting university in general (n = 23), and their excitement to have many experiences at university, despite having some worries still. Participants commented:

The summer school confirmed that I 10000% want to go to uni(versity) and maybe 25% made me think that I could live on campus alone.

I hope that I’ll eventually adapt to the new lifestyle and enjoy living in a new city.

Third, many participants highlighted their excitement to continue studying a subject that they enjoy during university (n = 19), and commented on their aspirations to achieve high grades academically, which sometimes was mentioned as the main motivating factor for going to university. Participants looked forward to:

… The chance to finally study something I am passionate about.

I just want to study psychology. I love the subject and whilst I understand the importance of other aspects of uni(versity) such as societies, that is the main thing I want.

Fourth, some participants also looked forward to gaining more independence (n = 15), confidence, and personal growth at university. Participants commented:

The freedom, independence, social experiences and the learning and work experience that will come with it are what I am looking forward to the most.

Being independent and as a result of becoming more confident.

… Trying to become more independent and grow as a person... become more outgoing, do well, and enjoy myself.

Fifth, a few students commented on the practical daily living aspects of university life (n = 5), such as staying in university accommodation. One student commented on looking forward to:

Having my own room, a place I can feel comfortable.

Finally, two students also commented on accessing support at university, such as going to tutors/lecturers for support, and “to receive help when I need it.”

## Discussion

This study aimed to evaluate a pre-transition programme, which was conducted annually over 5 consecutive years for autistic young people who are seeking to apply to and attend university. The main findings that emerged from the study were very positive. The programme was well-received by students who also reported a significant reduction in total and subscale scores on a questionnaire desired to measure concerns about university transition [Transition to University Questionnaire (TUQ)] following attendance at the programme. This is very positive when considering that the TUQ captured a broad range of concerns associated with university transition ranging from social difficulties, to academic challenges and time management, as well as daily living skills, thus suggesting that the autism summer school was considered by many students to be effective in alleviating a wide range of concerns associated with university life. Evaluation of participants’ quantitative and qualitative feedback also reiterated the usefulness of having a broad scope of different issues being discussed at the autism summer school, and that many students had a positive outlook and were optimistic about starting university after completing the programme.

The current study used the TUQ to evaluate a broad range of transition concerns associated with going to university, and seven factors or domains of concerns emerged from the factor analysis. The factors or domains of concerns identified in the current study largely match with the broad themes of transition challenges faced by autistic young people reported in the literature. First, four factors related to changes in the social environment and seeking support, as students move away from home (leave home; Factor 4), the need to make new friends (micro social world; Factor 1), to adapt to various social climates at university during daily living as well as for academic purposes (macro social world; Factor 3), and be able to seek appropriate sources of support (support; Factor 2) are consistent with the findings of qualitative studies.

The increasing social demands at university can be particularly challenging for autistic young people not only due to their social communication difficulties (Anderson et al. 2017; Knott and Taylor 2014; Longtin 2014; Ward and Webster 2018), but also because they often need to navigate the social scene more independently, without the support of their family. Although many autistic students recognise that socialisation with both peers and faculty staff at university is often necessary as part of the academic course and daily life, students often find such interactions exhausting (Van Hees et al. 2015), and can often misinterpret social information as well as being misunderstood by other people due to their social communication deficits (Gardiner and Iarocci 2014; Gelbar et al. 2014; Gobbo and Shmulsky 2014).

Even for autistic young people who report having friends, friendships are often not truly reciprocal as socialisation rarely takes place outside of predefined structured settings (Orsmond et al. 2004), and students often do not seek out these social partners for various sources of social support. This suggests that the functional aspects of social networks might be missing, even when there is some integrity in the structure of social networks (Bauminger and Kasari 2000). At university, poor social integration can be a major contributor towards feelings of isolation experienced by over 75% of university autistic students (Jackson et al. 2018), as well as feelings of anxiety and depression (Sarrett 2018; Van Hees et al. 2015; Ward and Webster 2018).

To help tackle some of the social challenges, many structured and unstructured social opportunities were built into the autism summer school programme to help facilitate participants to both initiate and take part in socialising with both typically developing students (the Ambassadors), autistic peers, and faculty staff. Many students commented on finding this diverse socialising experience to be particularly useful and helped to boost their confidence, and many were looking forward to new social opportunities that the university can help bring. In addition, the talk on the various clubs and societies that they can find at university also attracted the attention of some students at the prospects of having an opportunity to meet other people with shared interests. It is important to note that new social opportunities was the most highly endorsed response to what potential autistic students were most looking forward to about university. Clubs and societies may represent an invaluable avenue for enabling socialisation to occur.

The psychoeducation sessions containing material and practical exercises to help students identify and manage feelings of anxiety were also described as particularly helpful by a proportion of the students. This is in keeping with the high rates of anxiety problems reported by studies of autistic young people. Students with sub-clinical levels of anxiety may not have had this information previously, and support around such issues as managing anxiety is essential, which is reflected in this being the aspect of the autism summer school most frequently endorsed as helpful (see Table 6a).

Finally, to address how to best access support at university, the programme provided an informational talk about how to access the range of support formally available at university and the importance of disclosing your diagnosis. There were also small group, facilitated discussions about informal diagnosis disclosures, e.g., to new friends, flatmates, lecturers etc. Some students clearly enjoyed being able to discuss their diagnosis openly with others, whilst others who had previous negative experience of disclosure were more reticent in discussing the topic and less positive about the potential benefits of disclosure. A commonly reported experience was that of being treated differently by teachers and students, and a lack of control over who the information was shared with. In the UK, each university’s disability office facilitates tailoring transition plans to each student’s needs based on their disclosed diagnosis very early during the university application process. Absence of or late disclosure can therefore disrupt more effective transition planning, and can result in students unable to access the right type of support to meet their needs. Furthermore, within the university context, non-autistic peers often respond positively to disclosure (Brosnan and Mills 2016), and can be more understanding and supportive towards autistic students. For some students on the programme, finding out more about the pros and cons of disclosing one’s diagnosis with more in-depth information from multiple perspectives ahs enabled them to make more informed choices when choosing whether or not to disclose one’s diagnosis on university application forms.

Factor analysis of the TUQ also identified three domains of concerns that are non-social in nature, including academic challenges (Factor 5), time management (Factor 7), and daily living skills (Factor 6). All three domains correspond to the non-social challenges identified in the transition literature faced by autistic students (Anderson et al. 2017; Elias et al. 2017; Gelbar et al. 2014; Hewitt 2011; Longtin 2014), which is often linked to autistic students having poor executive function (Hewitt 2011; Ozonoff et al. 1991). Although the autism summer school did not offer any programmes directly aimed to improve executive function, a variety of measures were taken to help ease the many non-social concerns that students endorsed at baseline.

First, students received a lecture that focused on identifying and utilising their strengths [such as persistence, good attention to detail, the ability to systematise, and logical and rule-based thinking, e.g., (Baron-Cohen et al. 2009; Brosnan et al. 2017, 2016)] at university, and to help compensate for their weaknesses in planning and organising. This helped the students to feel more empowered, more confident about what they are capable of achieving, and to become more self-aware, which many students found to be very helpful. Increased self-awareness of personal strengths is of particular importance during transition to university, as better self-awareness can help students to self-advocate to receive more appropriate support at university (Dipeolu et al. 2014), and is a key factor in successful transition not only to university, but also to employment and throughout life (Kaffenberger 2011). Second, a key part of the current autism summer school programme is to fully immerse the student in independent living at university, thus including living in university accommodation on campus, and ordering food from university food outlets were central aspects. This element of practical daily living support proved to be very useful and well-received by many participants, who found the experience of successfully staying overnight at university to be very encouraging, and were looking forward to independent living at university by the end of the autism summer school.

It is important to note that the aim of the current autism summer school programme is to support and address concerns associated with transition to university for autistic students who are seeking to apply, or will be attending higher education. Although we do not directly address the issue of whether university may be the right choice for each student per se, by providing students with a sample of various aspects of what university life has to offer, we hope that the programme may indirectly help autistic students to more carefully consider what they might gain from university, and make a better informed decision as to whether higher education is the right choice for them. Considering alterative options following secondary education may be especially important for autistic students, especially if the ultimate aim is to help the autistic student gain independence, and attaining employment to ensure financial independence.

The rate of unemployment is particularly high autistic young people—with more than half (53.4%–60%) not having ever worked for pay since completing secondary education (Barneveld et al. 2014; Chiang et al. 2012; Roux et al. 2013). For those who are employed, the jobs are often low-skilled and poorly paid (Chiang et al. 2012; Roux et al. 2013). A systematic review of employment status in autism (Holwerda et al. 2012) found that the impact of educational attainment on employment is ambiguous, and a recent report in Germany found that 22.1% of 185 autistic adults felt overeducated in the jobs they’ve held for the longest time, and 31.3% felt overeducated in their current job (Frank et al. 2018). It is therefore necessary to take into consideration that higher education does not equate to employment, and continued support is needed during higher education to further enhance employment success for autistic students (Wehman et al. 2014).

Overall, the autism summer school identified concerns of potential autistic students who are thinking about attending university, and highlighted that these concerns can be significantly reduced through a pre-transitional autism summer school. Social, non-social, and anxiety-related concerns can be addressed with short pre-transitional programmes in a manner that potential autistic students find both helpful and enjoyable and enhance perceptions about successfully starting university.

### Limitations and Future Directions

Despite the many positive findings from the current evaluation of this university pre-transition programme, there are four important limitations to be considered. First, the factor analysis of the TUQ conducted in the current study is a preliminary and exploratory analysis, and the main purpose was to help evaluate whether changes in transition concerns following the transition programme can be observed on a finer scale, and across different domains of concerns, rather than just using the total score. It is important to note that therefore the internal consistency of some factors, such as support (Factor 2), was fairly low. Future research can seek to replicate the current factor analysis with a larger sample of autistic students, as well as compare whether the same factor structure can be found in a comparative group of typically developing students.

Second, the changes in transition concerns observed in the current study were only monitored for the concerns that each participant had endorsed prior to arriving at the autism summer school, rather than across all the worries of the TUQ. Although this selective evaluation helped to increase the practical feasibility of completing feedback measures within a limited time window on the final day of the autism summer school, it does not help to capture whether the autism summer school may have increased transition concerns of some students as they become more aware of what university life is truly like. Therefore, although it is positive to see many of the worries participants had about transition to university before they took part in the autism summer school were alleviated, it is important for future research to investigate whether other concerns that were not endorsed at baseline may have increased over time.

Third, it is important to take into consideration that the current evaluation failed to include the opinions of participants from 2014. However, given the similar demographic profile and magnitude of baseline worries of the students in 2014 compared to other year groups, it may be possible that the current findings are generalisable across the cohorts. An additional challenge was associated with the written nature of the evaluation feedback, as some students may have poor fine motor skills and therefore were reluctant to complete the qualitative feedback section because they were unable to write by hand. Future evaluations should seek to use multi-media technology and include a digitised version of the feedback form for the participant to complete online, therefore widening participation and increase equal access amongst the participants.

Finally, the current evaluation of the autism summer school transition programme only focused on short-term goals, such as reducing worries associated with going to university, and increasing a positive outlook on the transition process. However, little is known about the medium and long-term success of the programme, such as student enrolment status for participants who took part in the autism summer school compared to autistic students who did not partake in a transition programme, as well as long-term academic achievement, retention, and employment status after graduation. More longitudinal follow-up can help assess whether participants were able to utilise the different skills they have gained from summer school and use them flexibly to achieve medium and long-term goals, to evaluate whether any gains from the summer school are meaningful in real life. Future studies of transition programmes should therefore adopt a more longitudinal approach and aim to follow-up with participants over a longer period of time to evaluate how successful university transition is following the autism summer school, and how successful university transition may be further related to long-term quality of life for autistic young people.

We recommend that higher education institutions should offer tailored support to autistic students at all phases of university life i.e. pre-transition to university and while at university, on campus, and also include efforts to prepare students for life after graduation. First, we recommend that more institutions can provide similar transition programmes to autistic students who are either transitioning to, or considering applying to university. Having a structured programme that embeds key daily living components such as staying in university accommodation and ordering food from cafeterias can ease many of the worries associated with moving to university as reported by autistic students (Lambe et al. in press). Starting transition planning early can help identify clear goals and areas for skills development to facilitate a successful move to more independent living. It is also important to introduce students and families to opportunities available outside the realm of higher education, that might best suit the individual’s interests and skills. On campus, we recommend having more peer support for both social activities (such as through an Autism Social Group), and daily living and academic studies (such as having a buddy system/peer mentor). We also recommend increasing autism awareness and training for university staff. Such training should improve staff understanding about the challenges facing autistic students, as well as the high rates of co-occurring mental health issues. However, training should also place an emphasis on the strengths associated with autism, and how best to help students utilise these strengths and realise their potential at university. Finally, provision of practical training, and workshops supporting students to think about post higher education transitions into employment are also essential. These might include work experience placements and tailored application and interview skills training to ensure autistic students will be able to access the full range of benefits that can be associated with university education.

## Footnotes

1 Terminology: A recent study (Kenny et al. 2016) found that whereas professionals preferred to use person first language, referring to individuals as “people with autism”, members of the autism community prefer to use identity first language, referring to themselves as being “autistic”. In this paper, we choose to use identity-first language, and will refer to the students involved as “autistic students”.

2 Freshers’ week is the first week of university in the UK, prior to lecturers starting.
